# Delay to timely treatment in locally advanced cervical cancer: insurance inequities in access to gynecologic oncology

**DOI:** 10.1016/j.ajog.2025.02.032

**Published:** 2025-02-20

**Authors:** Jessica Liang, Audrey Mvemba, Megan Swanson, I-Chow (Joe) Hsu, Edwin Alvarez, Jocelyn Chapman, Katherine Fuh, Lee-may Chen, Stephanie Cham

**Affiliations:** School of Medicine, University of California San Francisco, San Francisco, CA; Department of Obstetrics and Gynecology, Kaiser Permanente, Oakland, CA; Division of Gynecologic Oncology, Department of Obstetrics and Gynecology & Reproductive Sciences, University of California San Francisco, San Francisco, CA; Division of Gynecologic Oncology, Department of Obstetrics and Gynecology & Reproductive Sciences, University of California San Francisco, San Francisco, CA; Division of Gynecologic Oncology, Department of Obstetrics and Gynecology & Reproductive Sciences, University of California San Francisco, San Francisco, CA; Division of Gynecologic Oncology, Department of Obstetrics and Gynecology & Reproductive Sciences, University of California San Francisco, San Francisco, CA; Division of Gynecologic Oncology, Department of Obstetrics and Gynecology & Reproductive Sciences, University of California San Francisco, San Francisco, CA; Division of Gynecologic Oncology, Department of Obstetrics and Gynecology & Reproductive Sciences, University of California San Francisco, San Francisco, CA; Division of Gynecologic Oncology, Department of Obstetrics and Gynecology & Reproductive Sciences, University of California San Francisco, San Francisco, CA

**Keywords:** cervical cancer, health disparities, insurance status, oncology, treatment delay

## Abstract

**BACKGROUND:**

Cervical cancer continues to disproportionately affect marginalized populations, with significant disparities in treatment and outcomes. Social determinants of health and insurance status have been associated with delays in treatment initiation, which can adversely affect clinical outcomes.

**OBJECTIVE:**

To determine the risk factors associated with delays in treatment initiation among patients with locally advanced cervical cancer and identify the time period of delay in treatment initiation.

**STUDY DESIGN:**

We conducted a retrospective cohort study of patients with locally advanced cervical cancer at a single institution between 2003 and 2023. The primary outcome was timely initiation of treatment, defined as treatment initiation within 60 days of diagnosis by biopsy. Multivariate analysis was used to assess the impact of insurance status and other demographic factors on treatment delays.

**RESULTS:**

Two hundred eighty patients were identified. The median time from biopsy to treatment initiation was 68.5 days (interquartile range, 52–104); 37.1% of patients received timely treatment initiation. Univariate analyses indicated patients with Medicaid had significantly increased odds of delayed treatment (odds ratio 2.76, 95% confidence interval 1.46–5.23) and living in a geographic location with a higher social vulnerability index (quartile 3 odds ratio 2.68 95% confidence interval 1.22–5.85). Multivariate analysis indicated that Medicaid was independently associated with delayed treatment compared to private insurance (odds ratio 2.42, 95% confidence interval 1.18–4.93). When time to treatment was stratified by time from biopsy to staging and time from staging to treatment start, delay was primarily attributable to time from biopsy to staging. In patients within the upper quartile of delay (>104 days), social risk factors including insurance-related issues and unmet social needs (eg, psychosocial distress, unstable housing, substance abuse) were identified as contributors to significant delays.

**CONCLUSION(S):**

Medicaid insurance was independently associated with lower odds of timely cervical cancer treatment, driven largely by delays between biopsy and staging. These findings underscore the need for targeted interventions to address insurance-mediated barriers to initiation of care, which may include screening and addressing unmet health-related social needs and social risks.

## Introduction

In 2024, there were approximately 14,050 projected new cases and 4290 projected deaths from cervical cancer in the United States.^1^ Cervical cancer is a preventable cancer, and access to screening and human papillomavirus vaccination has been associated with significantly reduced risk of diagnosis.^2^ However, marginalized populations have been shown to experience disproportionately higher rates of incidence and mortality, with significant disparities in treatment and outcomes identified along factors such as race, ethnicity, and rurality.^3-6^ Among the various challenges faced by individuals diagnosed with cervical cancer, navigating the journey from diagnosis to treatment is particularly demanding in an already at-risk population. This often requires complex care coordination including multiple providers, subspecialties, and facilities, particularly in patients with locally advanced cervical cancer (LACC).

Importantly, a spectrum of social needs including social determinants of health (SDOH) have been increasingly identified as important drivers in disparities in oncologic outcomes including cervical cancer.^7,8^ In a population-based cross-sectional study of New York City, neighborhood socioeconomic inequality was strongly associated with cervical cancer incidence rates, with the magnitude exceeding previous demographic associations such as race, ethnicity, or rurality.^4^ Furthermore, insurance has been shown to play a role in the receipt of high-quality care when evaluating a national database among all stages of treatment.^9^ Disparities in health-related social risks, such as transportation barriers or lack of a primary care provider, have also been associated with more advanced stage disease at time of diagnosis.^10^

Treatment of LACC can often be curative, but delay to treatment initiation results in prolonged time to symptomatic relief and potential impacts on clinical outcomes. Despite significant advances in cervical cancer prevention and treatment, the Society of Gynecologic Oncology notes delayed treatment as a major area of future work including addressing consequences on quality of life, which may be even greater in high-risk populations.^11^ While time to treatment has been shown to have a variable impact on survival, studies have shown an association with decreased disease control and lower survival in particularly profound delays of over 100 days.^12-14^ Prior work suggests utilizing 60 days from diagnosis to receipt of primary treatment initiation as a benchmark guideline for quality care in LACC tumors.^9^ Due to the potential prognostic impact of time to treatment, the European Society of Gynecologic Oncology (ESGO) and European Society for Radiotherapy and Oncology have set an even more stringent quality indicator of 42 days as the benchmark for timely treatment.^15^ Thus, identifying risk factors for delayed time to treatment may help determine those in need of further assistance in this transitional period and identify mechanisms to improve quality of life and clinical outcomes in this high-risk population.^8^

The purpose of this study was to deeply characterize delays to timely treatment, which is typically not readily available in routine cancer databases. Our primary objective was to determine risk factors associated with receipt of timely treatment in this population. Our secondary objectives included determining the stage of treatment initiation that was delayed (biopsy to staging vs staging to treatment initiation) and characterizing reasons for delay.

## Materials and methods

We conducted a retrospective single-institution cohort study of patients diagnosed with LACC who received care at the University of California, San Francisco (UCSF) between January 1, 2003 and September 30, 2023. Inclusion criteria consisted of a International Federation of Gynecology and Obstetrics 2018 Stage IB3-IVA cervical cancer diagnosis, receipt of curative or palliative cancer-directed treatment, and age 18 years or older. We excluded those with insufficient follow-up records with our department, a remote initial history of cervical cancer not managed at UCSF, actively undergoing treatment at the time of data abstraction, those with rare histology (eg, neuroendocrine), and those who did not receive cancer-directed therapy. This study was approved by the UCSF institutional review board.

The primary outcome was timely initiation to treatment, classified as 60 days or less from diagnosis to treatment initiation, as suggested as a benchmark for quality care based on prior work evaluating insurance status disparities in cervical cancer in an analysis of the National Cancer Database (of note, a more stringent benchmark of 42 days has been established by international colleagues in the ESGO and European Society of Radiation Oncology).^9^ Treatment initiation was defined as the first day of curative or palliative cancer-directed treatment (eg, chemoradiation or palliative radiation). Secondary outcomes included time from biopsy to staging and time from staging to treatment to identify the time period where delays were occurring. Timing of staging was determined based on a hierarchy, first using the date of biopsy of distant disease (eg, lymph nodes). If a biopsy was not completed, the date of radiology imaging (eg, positron emission topography, magnetic resonance imaging) was used. If neither biopsy nor imaging was available, the date of exam under anesthesia for clinical staging was used.

Demographic risk factors abstracted included age, self-reported race, insurance status, travel distance, and Charlson Comorbidity Index (CCI), and social vulnerability index (SVI). Travel distance and time were determined by averaging estimates in Google Maps at a uniform arrival time and day of a typical clinic visit (eg, 10:00 am, Monday December 11th, 2023) into quartiles. SVI is a census tract-based index that utilizes fifteen variables that evaluate socioeconomic status, household composition and disability, minority status and language, and housing type and transportation into account as a proxy for individual-level socioeconomic status. SVI was abstracted by cross-referencing patients’ listed home address with the Agency for Toxic Substances and Disease Registry.^16^ Oncologic factors abstracted included stage and histology. In patients in the upper quartile of delayed time to treatment, we performed an in-depth review of all available records from social work, nursing, or provider notes, and any other available outside records to identify reasons for potential delay.

Fisher’s exact tests and Kruskal-Wallis tests were used to determine demographic and oncologic risk factors associated with timely treatment. We performed univariate and multivariate logistic regression analysis using significant covariates (*P*<.20) from the univariate analysis. We performed backward selection to create a parsimonious set of variables thought to have clinical implications to create the multivariate model (excluding age and CCI in the final model). Due to the relatively high number of missing values with difficulty in meaningful interpretation, the final multivariable model excluded race and ethnicity as a covariate. Assumptions were tested with both goodness of fit and evaluated for collinearity. Given the long time period of data collection and potential impact of the COVID pandemic a sensitivity analysis excluding patients diagnosed between January 1, 2020, and December 31, 2021, was performed. Data analysis was performed using STATA 18 (StataCorp LLC, College Station, TX). *P*<.05 was considered statistically significant.^17^

## Results

**FIGURE 1:**
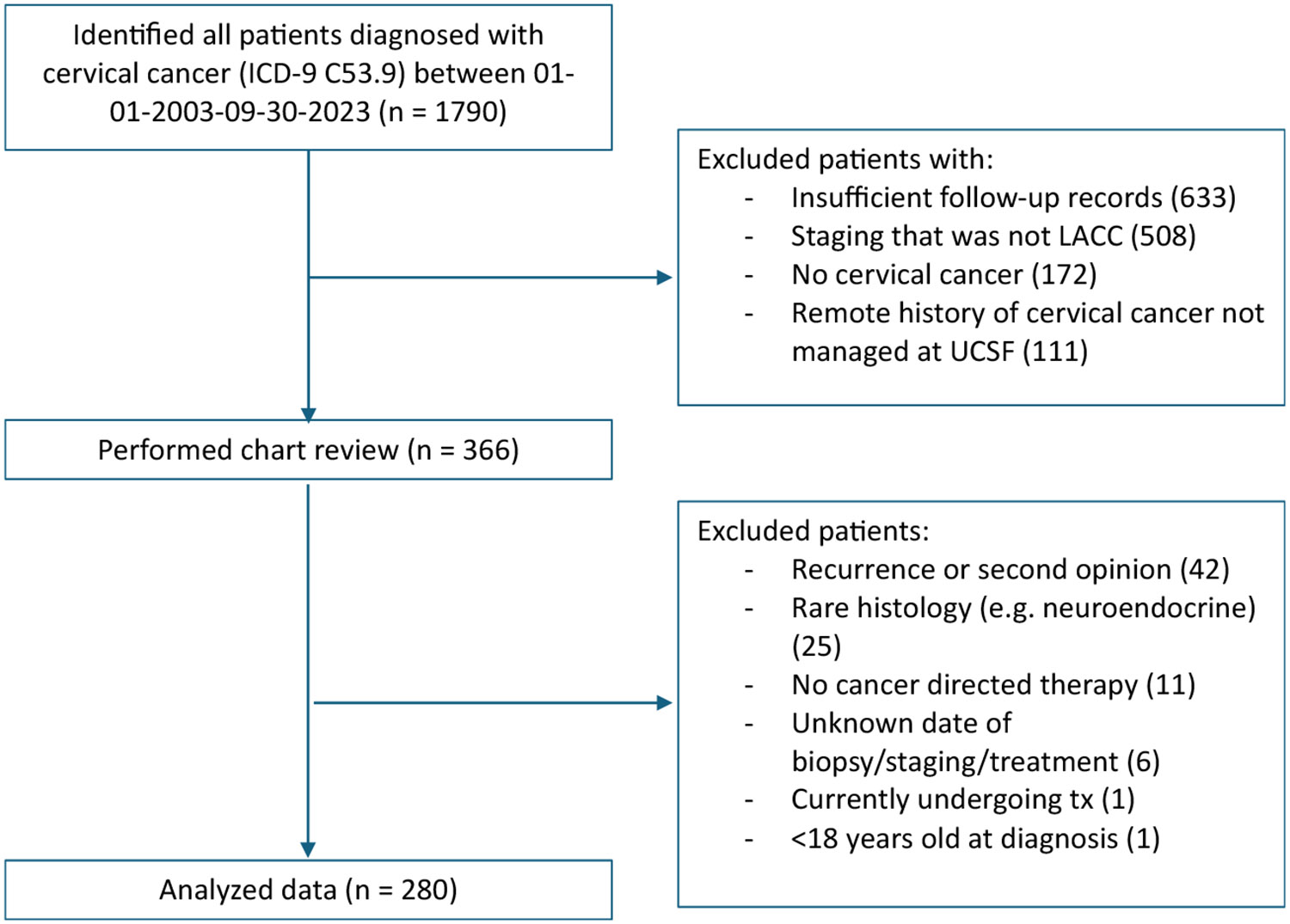
Consort diagram of cohort selection

A total of 280 patients were identified for this study, and a CONSORT diagram for cohort selection can be seen in Figure 1. Patient characteristics stratified by timely versus delayed treatment initiation can be seen in Table 1. One hundred four (37.1%) received timely initiation to treatment. The median time from biopsy to treatment initiation was 68.5 days (interquartile range [IQR], 52–104).

Of the study population, 49.6% self-reported as White, 30.4% had no self-reported race listed; 31.4% were 40 to 49 years old; 75.4% of patients had Medicaid insurance, 17.1% had private, 6.4% had Medicare, and 1.1% were uninsured; 65.4% of patients were stage III, and 78.2% were squamous cell carcinoma histology; 36.9% of patients with timely treatment had no self-reported race compared to 19.2% of patients with delayed treatment had no self-reported race; 81.8% of patients with timely treatment were on Medicaid compared to 64.4% of patients with delayed treatment; 24.4% of patients with delayed treatment were in the third highest SVI quartile compared to 14.4% of patients with timely treatment.

Univariate analyses indicated that patients with Medicaid had significantly increased odds of delayed treatment (odds ratio [OR] 2.76, 95% confidence interval [CI] 1.46–5.24) compared to private insurance (reference). Patients in the third highest quartile of SVI also experienced increased odds of delayed treatment (OR 2.68, 95% CI 1.22–5.85) compared to patients in the first SVI quartile (least vulnerable, reference). Further, patients with stage IIIA-C cancer experienced decreased odds of delayed treatment (OR 0.56, 95% CI 0.31–0.99) compared to patients with stage IB3-IIB cancer (reference). Patients with no self-reported race were also found to have increased odds of delayed treatment (OR 2.62, 95% CI 1.43–4.78) compared to White patients (reference) and not Hispanic or Latino were found to have decreased odds (OR 0.53, 95% CI 0.30–0.02) compared to Hispanic or Latino (reference). Age 70 years and older was associated with decreased odds (OR 0.19, 95% CI 0.05–0.79) compared to younger patients less than 30 years old (reference). Travel time, CCI, and histology were not significantly associated with odds of delayed treatment (Table 2).

Multivariate analysis indicated Medicaid was independently associated with 2.42 times increased odds of delayed treatment (95% CI 1.18–4.93) compared to private insurance (Table 3). SVI and stage were not found to be significant in multivariate analysis.

**FIGURE 2:**
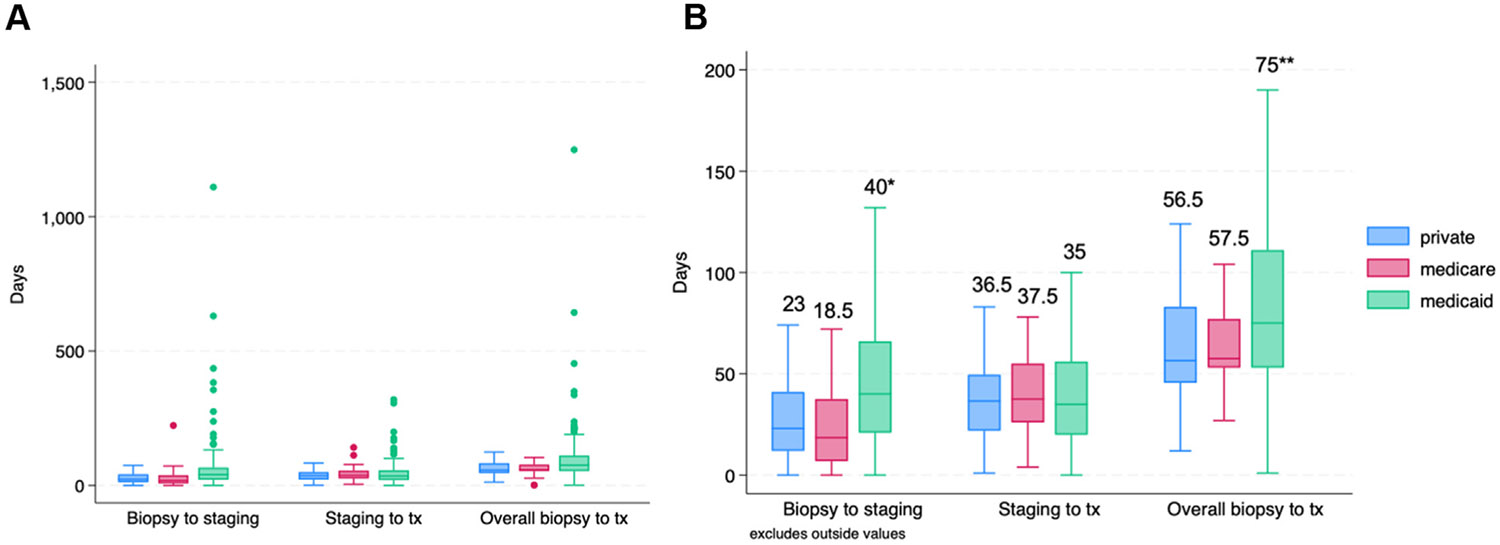
Time to treatment by components and insurance type Comparison of insurance type and time to treatment and its components of time from biopsy to staging and time from staging to treatment in (**A**) all patients in the cohort (**B**) excluding outliers with extreme delays. **P<.002 (green), *P<.001 (blue), Kruskal-Wallis test.

The median time from biopsy to staging for the whole cohort was 35 days (IQR, 15–59). The median time from staging to treatment initiation was 35 days (IQR, 20.5–54.5). Patients with Medicaid insurance experienced significantly longer delays of time from biopsy to treatment start compared to private insurance (median 56.5 vs 75 days, *P*<.002). When time to treatment was stratified by time from biopsy to staging and time from staging to treatment start, delay comparing Medicaid to private insurance was primarily attributable to time from biopsy to staging (median 23 vs 40 days, *P*<.001) (Figure 2).

For patients in the upper quartile of delay (>104 days), we evaluated all available notes from various clinicians and providers (eg, physician, nursing, social work) and identified reasons stated in the notes that were indicated as possible associations with delay (Table 4). Of the 70 patients in the upper quartile, 14 (20.0%) had insurance-mediated delays (eg, insurance authorization, difficulties with switching insurance), 10 (14.3%) experienced delay due to social risks and needs (eg, expired visas, unstable housing, unreliable transportation, psychosocial stressors), 4 (5.7%) experienced anxiety surrounding treatment, 4 (5.7%) had delays due to pregnancy, 4 (5.7%) had delays due to emergency department visits and medical comorbidity complications, 3 (4.2%) had difficulty scheduling with medical providers, and 1 (1.4%) was experiencing incarceration at the time of diagnosis. Twenty four (34.3%) had no documented reason for delay.

A sensitivity analysis excluding patients possibly affected by the COVID pandemic (diagnosis between January 1, 2020, and December 31, 2021, N=38) found outcomes were relatively unchanged. The median time to treatment was 68 days (IQR 52–105) and the rate of timely initiation continued to remain very low at 37.2%. Multivariate modeling continued to support that Medicaid insurance was the greatest independent predictor of delayed initiation to treatment (OR 2.71, 95% CI 1.03–5.26).

## Comment

### Principal findings

In our study, only 37.1% of patients received timely treatment for LACC. The median time from biopsy to treatment initiation was 69 days with over one-fourth of patients experiencing delays of 104 days or more. Our analysis indicated patients with Medicaid insurance, no self-reported race, staging at IB1-IIB, and medium-high SVI (third quartile) were significantly less likely to receive timely treatment initiation. In multivariate analysis, Medicaid insurance was independently associated with delay to treatment. This delay was found to be primarily driven by a prolonged time between biopsy and the completion of staging. Our analysis of the upper quartile of patients with significant delays of over 104 days indicated multiple social risk factors including insurance-mediated delays, social risks and needs, and anxiety surrounding treatment.

### Results in the context of what is known

Our study further supports the relationship between insurance status and delay to treatment in cervical cancer, and here we provide further insights on the specific components and risks for delay. In an analysis of the National Cancer Database, Medicaid and uninsured patients were significantly less likely to receive timely initiation of treatment for both early stage and LACC and were associated with lower overall survival.^9^ In this study, we examined a diverse set of risk factors including SVI for delayed treatment and identified Medicaid insurance as an independent risk factor. The relationship between SVI and delays to cancer treatment noted in this study mirrors that of other studies, as previous work has shown an association between cervical cancer incidence, colorectal cancer morbidity, and lung cancer mortality rates and neighborhood SVI.^4,18-20^ This is particularly significant, as studies have found links between prolonged delays to cancer treatment and higher rates of tumor progression, decreased treatment efficacy, and worsening survival outcomes.^21^ Of note, while prior studies have demonstrated that the 2014 expansion of Medicaid increased coverage, examination of national data indicated expansion was found to be associated with worse 2-year survival in cervical cancer.^22^ Our work further supports the need to evaluate specific reasons and identify potential interventions for inequities in cervical cancer care associated with receipt of Medicaid insurance.

Our data indicated the primary delay was the time between biopsy and staging, potentially identifying insurance-mediated delays to establishment of gynecologic oncology care as the primary barrier. These findings are supported by a smaller single-institution study of 72 patients, which found patients on Medicaid experienced a longer time interval between diagnosis and initial appointment with a radiation oncologist.^23^ Medicaid insurance has been associated with up to 44% longer wait times for an appointment with an obstetrics and gynecology subspecialist compared to commercially insured patients.^24^ Further, comprehensive community cancer programs were found nationally to be significantly less likely to provide access to care for patients with Medicaid.^25^ Prior research in evaluating time to treatment in lung cancer identified a benchmark of 52 days from diagnosis to treatment and highlighted that a majority of avoidable delays could have possibly been avoided through optimizing clinical transitions and follow-up to care.^26^ Thus, access and transitions of care to gynecologic oncology subspecialists for this already vulnerable population appear to be an important inflection point.

### Clinical implications

While previous studies have characterized the relationship between SDOHs (ie, rurality, race/ethnicity, and neighborhood) and delays to treatment, individual social risks and needs may also play a significant role in transitions of care. Insurance status is often a reflection of social risks such as socioeconomic status and occupation, and we identified in our study that many Medicaid recipients have significant related social risks. Unlike SDOHs, which represent environmental conditions, social risk factors are individual psychosocial elements that directly impact health outcomes and may thus be altered through intervention. Our results highlight the role of insurance as both an independent risk factor and a reflection of social risks that contribute to delays in treatment. Acknowledging this, interventions that address insurance-mediated barriers on both individual and systemic levels are necessary to mitigate these disparities. Patients with cervical cancer are often diagnosed by primary care providers or gynecologists, who then must refer them to gynecologic oncologists for specialized treatment. Our findings indicate that this transition is a key step where delays occur, and these delays are likely compounded by systemic barriers such as insurance issues and individual social risks. Routinely identifying health-related social needs, improving accessibility, and close monitoring of these referral systems during this transition may play a critical role in mitigating these delays and improving patient outcomes.

### Research implications

Future directions include studying interventions to improve referral and navigation processes for patients with health-related social needs and SDOH such as Medicaid recipients. One of the first and basic steps includes routine social screening to identify individual patients that may be more susceptible to social risk. A single institution study of an urban academic gynecological oncology clinic that implemented routine basic resource screening was able to successfully provide resources to 69% of patients who screened positive and requested assistance.^27^ A hospital wide systems interventional study that evaluated the role of utilizing lay navigators in the older adult population with newly diagnosed cancer noted a significant decrease in total Medicare costs and healthcare utilization, indicating that this methodology may be beneficial for Medicaid recipients as well.^28^ Further, a multisite patient navigation intervention for patients with cervical or breast cancer resulted in a significant decrease in time to diagnosis for navigated subjects.^29^ We identified that some of the associated delays can be attributed to delays in obtaining Medicaid insurance, a barrier that may be mitigated by routine SDOH screening and patient navigation intervention as well. Another scalable approach to increasing access to care includes increasing accessibility to telemedicine visits with a gynecologic oncologist, or “teleoncology,” which provides opportunities for outreach to both patients and their local oncology providers in underserved areas and decreases the financial burden of care.^30,31^ On a systemic level, advocacy work to modify state policies to include social risk-adjusted payments for Medicaid recipients who typically require more navigation and social resources is needed to support make these efforts financially feasible and may incentivize comprehensive community cancer programs to provide care to Medicaid patients and expand access to quality cancer care.^25^

### Strengths and limitations

While our study was strengthened by its diverse patient population, we recognize several important limitations. Our study utilized SVI, which only provides a geographic composite measure based on census data and may not be applicable to the individual patient. The quartiles of SVI had wide confidence intervals in univariate analysis and were not powered to detect conclusions generated from this smaller study. Secondly, approximately 37% of self-reported race data was not available. Many patients were also lost to long-term follow-up, and this study was underpowered to determine the clinical implications of progression and survival. This study was also performed at a tertiary academic care center and many patients were excluded due to insufficient records (eg, received or initiated treatment elsewhere in the community) thus these data may not be reflective of outcomes in all practice settings. However, in this context, this may bias the data to underreport potential delays to treatment initiation. This study was also performed over a longer period of time, but a sensitivity analysis excluding patients during potential major changes (eg, COVID pandemic) showed little change in the primary outcome. However, we were able to access detailed annotation of reasons for delay to care through our patient records, and thus were able to deeply characterize individual patient experiences in a way that is not typically available in larger data sets. Through this, we were able to include important data regarding social risk that is often missing from the literature. Understanding these specific factors contributing to delays is crucial in developing targeted interventions.

## Conclusions

In summary, we identified significant delays in treatment initiation among cervical cancer patients with Medicaid insurance, which were primarily driven by prolonged time between biopsy and the completion of staging procedures, highlighting insurance-mediated barriers to care. Our findings emphasize the need for future studies to examine associations with clinical outcomes and identify meaningful interventions that target the referral and navigation process (eg, patient navigators, community health workers, routine SDOH screening) and provide system-level resources (eg, reimbursement for additional time and resources expended) to address social needs to reduce this inequity in delivery of care, particularly for patients with Medicaid insurance and those from socially vulnerable backgrounds.

## Figures and Tables

**TABLE 1:** Characteristics of the patient cohort comparing timely vs delayed treatment initiation

Total cohort	Overall	Timely treatment (<60 d)	Delayed treatment (>60 d)	*P* value
N	280	104 (37.1%)	176 (62.9%)	
Age				.112
<30	16	4 (3.8%)	12 (6.8%)	
30–39	64	22 (21.2%)	42 (23.9%)	
40–49	88	28 (26.9%)	60 (34.1%)	
50–59	61	24 (23.1%)	37 (21.0%)	
60–69	29	12 (11.5%)	17 (9.7%)	
≥70	22	14 (13.5%)	8 (4.5%)	
Race				.002^a^
American Indian/Alaskan native	5	1 (1.0%)	4 (2.3%)	
Asian/Pacific Islander	33	11 (10.6%)	22 (12.5%)	
Black or African American	11	4 (3.8%)	7 (4.0%)	
White	139	62 (59.6%)	77 (43.8%)	
More than 1	7	6 (5.8%)	1 (0.6%)	
Unknown	85	20 (19.2%)	65 (36.9%)	
Ethnicity				.070
Hispanic or Latino	87	24 (23.1%)	63 (35.8%)	
Not Hispanic or Latino	184	77 (74.0%)	107 (60.8%)	
Unknown/not reported	9	3 (2.9%)	6 (3.4%)	
Insurance status				.002^a^
Private	48	27 (26.0%)	21 (11.9%)	
Medicare	18	10 (9.6%)	8 (4.5%)	
Medicaid	211	67 (64.4%)	144 (81.8%)	
Uninsured	3	0 (0.0%)	3 (1.7%)	
SVI quartile				.099
1 (least vulnerable)	58	28 (32.6%)	30 (20.7%)	
2	58	21 (24.4%)	37 (25.5%)	
3	58	15 (17.4%)	43 (29.7%)	
4 (most vulnerable)	57	22 (25.6%)	35 (24.1%)	
Distance from facility (time, quintiles)				.931
<45 min	66	22 (22.7%)	44 (26.2%)	
45-<90 min	62	24 (24.7%)	38 (22.6%)	
90-<135 min	70	26 (26.8%)	44 (26.2%)	
≥135 min	67	25 (25.8%)	42 (25.0%)	
Stage				.131
IB3-IIB	79	22 (21.2%)	57 (32.4%)	
IIIA-IIIC	183	75 (72.1%)	108 (61.4%)	
IVA	18	7 (6.7%)	11 (6.2%)	
Charlson comorbidity index (median)				.082
≤6 (low)	159	52 (50.0%)	107 (60.8%)	
≥7 (high)	121	52 (50.0%)	69 (39.2%)	
Histology				1.000
Squamous cell carcinoma	219	81 (77.9%)	138 (78.4%)	
Adenocarcinoma	61	23 (22.1%)	38 (21.6%)	

*SVI*, social vulnerability index.

a *P*<.05.

**TABLE 2:** Univariate analysis of factors associated with delayed treatment (>60 d)

Risk factor	OR (95% CI)	*P* value
Age		.107
<30	Reference	
30–39	0.64 (0.18–2.21)	
40–49	0.71 (0.21–2.41)	
50–59	0.51 (0.15–1.78)	
60–69	0.47 (0.12–1.82)	
≥70	0.19 (0.05–0.79)^a^	
Race		.002
White	Reference	
Asian/Pacific Islander	1.61 (0.73–3.57)	
Black or African American	1.41 (0.39–5.03)	
American Indian/Alaskan Native	3.22 (0.35–29.56)	
More than 1	0.13 (0.02–1.14)	
Unknown	2.62 (1.43–4.78)^a^	
Ethnicity		.014
Hispanic or Latino	Reference	
Not Hispanic or Latino	0.53 (0.30–0.92)^a^	
Unknown	0.76 (0.18–3.29)	
Insurance status		.002
Private	Reference	
Medicare	1.03 (0.35–3.06)^a^	
Medicaid	2.76 (1.46–5.24)^a^	
Uninsured	1	
SVI quartile (higher more vulnerable)		.021
1 (least vulnerable)	Reference	
2	1.64 (0.78–3.46)	
3	2.68 (1.22–5.85)^a^	
4 (most vulnerable)	1.48 (0.71–3.12)	
Distance from facility (time, quintiles)		.930
<45 min	Reference	
45-<90 min	0.79 (0.38–1.63)	
90-<135 min	0.85 (0.42–1.71)	
≥135 min	0.84 (0.41–1.71)	
Stage		.121
IB3-IIB	Reference	
IIIA-IIIC	0.56 (0.31–0.99)^a^	
IVA	0.61 (0.21–1.76)	
Charlson comorbidity index (median)		.078
≤6 (low)	Reference	
≥7 (high)	0.64 (0.40–1.05)	
Histology		.918
Squamous cell carcinoma	Reference	
Adenocarcinoma	0.97 (0.54–1.74)	

*CI*, confidence interval; *OR*, odds ratio; *SVI*, social vulnerability index.

a *P*<0.05.

**TABLE 3:** Multivariate analysis of factors associated with delayed treatment (>60 d)

Risk factor	OR (95% CI)
Insurance status	
Private	Reference
Medicare	1.05 (0.28–3.96)
Medicaid	2.42 (1.18–4.93)
Uninsured	1
SVI quartile (higher more vulnerable)	
1 (least vulnerable)	Reference
2	1.50 (0.69–3.28)
3	2.10 (0.91–4.86)
4 (most vulnerable)	1.27 (0.58–2.80)
Stage	
IB3-IIB	Reference
IIIA-IIIC	0.58 (0.31–1.11)
IVA	0.75 (0.23–2.45)

*SVI*, social vulnerability index.

**TABLE 4:** Reasons cited in detailed chart review of cited reasons for delays to treatment in the upper quartile of cohort (>104 d)

N=70	%	Examples cited in chart review
Not documented	34.3%	
Insurance-mediated delay	20%	“not seen for over a year due to delay in her insurance” “ongoing issues with her insurance” “hospital does not take patient insurance” “patient’s insurance denied her upcoming surgery”
Social risks/needs	14.3%	“visa expired, had to return to [home]” “psychosocial issues (homelessness, polysubstance abuse, bipolar disorder)” “prefers to be in [home country] because she is lonely here”
Anxiety/fear	5.7%	“sexually abused as a child and does not tolerate pelvic exams… contributed to her decision to delay her treatment until symptoms became unbearable” “delaying chemotherapy because she remembers the way she felt when she was first diagnosed”
Pregnancy	5.7%	“terminated pregnancy”
Follow-up showed progression	5.7%	“planned hysterectomy was aborted secondary to intraoperative findings of nodal metastases”
Emergency department visits and/or other medical comorbidities	5.7%	“delayed due to pancytopenia” “ED admission due to pain and cellulitis”
Difficulty contacting provider	4.2%	“delay due to difficulty contacting rad/onc for an appointment”
Incarceration	1.4%	“urged to seek treatment once released from prison”

## AJOG at a Glance

### Why was this study conducted?

To identify risk factors associated with delays in treatment initiation for locally advanced cervical cancer.

### Key findings

Medicaid insurance was independently associated with delays in treatment initiation, primarily due to prolonged time between biopsy and staging. Medicaid recipients in our study were found to experience delays mediated by both insurance and social risks.

### What does this add to what is known?

This study highlights insurance status as a key driver of treatment delays in cervical cancer. It emphasizes the need for targeted interventions of modifiable health-related social needs associated with delayed treatment initiation.
